# Advanced Lipid Technologies® (ALT®): A Proven Formulation Platform to Enhance the Bioavailability of Lipophilic Compounds

**DOI:** 10.1155/2019/1957360

**Published:** 2019-07-08

**Authors:** Miguel A. Lopez-Toledano, Vaibhav Saxena, Jason D. Legassie, Haiyang Liu, Ajay Ghanta, Stephen Riseman, Courtney Cocilova, Ahmed Daak, Thorsteinn Thorsteinsson, Adrian L. Rabinowicz, Frederick D. Sancilio

**Affiliations:** ^1^Micelle BioPharma, Inc. (Former Sancilio & Company, Inc., SCI), 3874 Fiscal Court, Riviera Beach, FL, 33404, USA; ^2^Former Sancilio & Company, Inc. (SCI), USA

## Abstract

Despite recent advances, the drug development process continues to face significant challenges to efficiently improve the poor solubility of active pharmaceutical ingredients (API) in aqueous media or to improve the bioavailability of lipid-based formulations. The inherent high intra- and interindividual variability of absorption of oral lipophilic drug leads to inconsistent and unpredictable bioavailability and magnitude of the therapeutic effect. For this reason, the development of lipid-based drugs remains a challenging endeavour with a high risk of failure. Therefore, effective strategies to assure a predictable, consistent, and reproducible bioavailability and therapeutic effect for lipid-based medications are needed. Different solutions to address this problem have been broadly studied, including the approaches of particle size reduction, prodrugs, salt forms, cocrystals, solid amorphous forms, cyclodextrin clathrates, and lipid-based drug delivery systems such as self-emulsifying systems and liposomes. Here, we provide a brief description of the current strategies commonly employed to increase the bioavailability of lipophilic drugs and present Advanced Lipid Technologies® (ALT®), a combination of different surfactants that has been demonstrated to improve the absorption of omega-3 fatty acids under various physiological and pathological states.

## 1. Introduction: Fat Absorption, Bioavailability, and Food Effect

Most dietary fatty acids are consumed in the form of triglycerides, an ester formed from glycerol and three fatty acid groups. However, most nutritional and pharmaceutical fatty acid products, particularly omega-3, are developed in ethyl ester (EE) form, aiming to remove industrial contaminants (e.g., heavy metals, dioxins) and increasing the quality of the final product. EEs are purified by molecular distillation techniques (low temperature vacuum distillation) or, in more recent years, by chromatography [1–3]. The few purified fatty acids available as triglycerides in the market are synthesized by converting the purified ethyl ester back to the triglyceride form after purification. In either form, fatty acid bioavailability will depend on the hydrolysis of the ester bond associated with the glyceride or ethyl ester moiety. In the human digestive tract, this de-esterification is performed primarily by human gastric lipases (HGL) and human pancreatic lipases (HPL) [4, 5]. All lipids are hydrophobic substances that are highly insoluble in aqueous media, where they tend to form large fat globules. In the digestive tract, the water soluble HPL will only be enzymatically active at the surface of these large fat globules. Therefore, the enzymatic activity of the lipases is inversely correlated with the size of the fat globules.

Bile salts are fundamentally steroid acids synthesized by the liver, conjugated with sodium and potassium salts of glycine and taurine and then stored in the gallbladder. They are released into the bile duct when dietary fat is detected. Bile salts are amphipathic molecules, with both hydrophobic and hydrophilic regions. They play a crucial role in lipid digestion by breaking up large fat globules into smaller droplets in a process known as emulsification. Emulsification greatly increases the surface area of the lipid globules, which allows greater access of HPL to the lipids and improves the de-esterification efficiency. After hydrolysis of either triglycerides or EE, the resulting monoglycerides and free fatty acids will associate with bile salts and phospholipids to form micelles. The International Union of Pure and Applied Chemistry (IUPAC) defines a micelle as a particle of colloidal dimensions that exists in equilibrium with the molecules or ions in solution from which it is formed [6]. In the digestive tract, micelles are formed by bile salts and lipids, forming aggregates with the hydrophilic “head” regions in contact with surrounding aqueous environment, sequestering the hydrophobic single-tail regions in the micelle center. These micelles facilitate the transport of the monoglycerides, free fatty acids, fat-soluble vitamins, cholesterol, and other lipophilic compounds to the surface of enterocytes lining the digestive track, facilitating their absorption [7, 8].

The efficiency of the emulsification in the digestive tract is highly dependent on bile salts, which are only released into the intestine by the gallbladder when dietary fat is detected at reasonably high levels [9]. When a low amount of dietary fat is present, the release of bile salts does not occur and thereby lowers the efficiency of fat emulsification and hydrolysis, resulting in a reduction of fatty acid bioavailability (BA). The impact of diet on the BA of nutrients or drugs is called “food effect” [10].

The absorption and BA of dietary fats and any other kind of lipophilic compounds are likely to be significantly impacted in the absence of food or low fat in the diet and remain one of the major barriers for developing lipid-based treatments with high and consistent bioavailability.

These differences in BA are applicable not only for natural lipids found in the diet (i.e., vitamins A, D, E, and K), but also for oral drug products with API exhibiting poor water solubility. In fact, a growing number of drugs (40% of approved drugs and almost 90% of developmental pipeline drugs) have poor biopharmaceutical properties [11]. Poorly water-soluble drugs may be subjected to changes in BA resulting from the amount of dietary fat present when the substance is consumed. An inconsistent bioavailability leads to higher differences between individuals, which may not only have differentiation and unpredictability in clinical effects on individuals, but can also significantly impact the study design and result evaluation of clinical trials. In these cases, a larger sample size is needed to reach statistical significance, increasing the cost of the study, the complexity of clinical result analysis and interpretation, and the risk of the drug failing in Phase 3 clinical studies. Therefore, effective strategies to assure a predictable, consistent, and reproducible BA for lipid-based medications are needed.

## 2. Strategies to Overcome Impaired Bioavailability of Lipophilic Drugs

The poor BA observed with lipophilic medications is one of the major hurdles in drug development, due to limited or inconsistent API solubility. Some of the common approaches to solve this problem are briefly reviewed below.

Approaches to increase API solubility for solid oral dosage forms have traditionally focused on particle size reduction, prodrugs, salt forms, and cocrystals. Particle size reduction increases the surface area of a crystalline drug to allow for more rapid dissolution and increased solubility kinetics. Methods for reducing API particle size to micrometer and even nanometer size are well documented and widely available [12, 13]. Prodrugs are commonly used to improve the solubility, permeability, and BA of challenging APIs [14–16]. A prodrug, as defined by IUPAC, is a drug that undergoes biotransformation (metabolism) before exhibiting a pharmacological effect. A few of the common biotransformable function groups that are used to create prodrugs are carbonates, esters, amides, carbamates, ethers, and phosphates. If an API has an ionizable moiety, then a salt form with different counter ions can have dramatic effects on drug solubility and chemical stability [17–19]. More recently the approach of utilizing cocrystals has gained popularity for increasing drug solubility. Cocrystals are formed when an API is stoichiometrically complexed, nonionically, in a crystal with a coformer (usually an organic acid or another Generally Recognized as Safe (GRAS) excipient) that reduces the crystal lattice energy and greatly increases the solubility of the drug. Cocrystals can be formed even if the API lacks an ionizable function group and can be designed to have multiple advantages including improved processability, increased stability, and most importantly increased solubility [20, 21]. An added benefit to the cocrystal approach is that the resultant drug is treated like an API polymorph from an FDA regulatory perspective [22]. This differs from different salt forms or prodrugs which are considered new APIs and require significant additional development work before regulatory filing and approval.

Other common avenues to increase lipophilic drug BA involve using solid amorphous forms, cyclodextrin inclusion complexes, and lipid based drug delivery systems including self-emulsifying systems and liposomes. Amorphous forms of a drug lack defined crystal lattices, which greatly increases the drug solubility as high lattice energy is a barrier for drug solubility and dissolution [23]. Common approaches to amorphous drug formation are hot melt extrusion [24] and spray-drying [25]. The main drawback to amorphous forms is the tendency of the drug to revert to the more stable crystalline form slowly over time potentially leading to reduced drug solubility. Cyclodextrins are used to increase the solubility and BA of lipophilic compounds by forming inclusion complexes by trapping the drug in their lipophilic interior cavity. The exterior of cyclodextrins, which are composed of glucopyranose units, are water soluble which then allows the lipophilic drug to be easily solubilized as a complex. The drug complex can then be dosed orally where it acts to solubilize and deliver the lipophilic drug [26, 27]. Lipid based drug delivery systems in their simplest form involve dissolving the lipophilic API in a lipid carrier [28, 29]. These simplest formulations tend to suffer from high food effects, as the lipid carrier needs bile salts to become properly dispersed. Additional ingredients such as cosolvents and surfactants can be added that enable the formulation into a Self-Emulsifying Drug Delivery System (SEDDS). These systems have the potential to spontaneously form emulsions, microemulsions, nanoemulsions, or micelles when the dosage form interacts with the stomach contents [30–33]. As these formulations are self-dispersed, they tend to have lower demonstrated food effects and because of the liquid or semisolid state, these formulations are usually encapsulated in and delivered by hard or soft gelatin capsules. Some limitations of solubilization and SEDDS are API solubility, API propensity to precipitate in the dosage form or in the gastrointestinal tract, and increased degradation potential of the API in the dissolved state. Liposomes have also been used to improve lipophilic BA. Liposomes are similar to the emulsions described previously in that they are composed of small emulsion like droplets except they employ phospholipids to form the dispersion and they are formed from a bilayered arrangement of the phospholipids [34, 35]. When they are used for lipophilic drug delivery, the lipid is contained within the lipophilic region of the bilayer, which limits the drug loading capacity. Liposomes are not known to form spontaneously and require energy to be formed. Once formed in a water based system, they are typically lyophilized and dosed in hard shell capsules or reconstituted in aqueous media right before dosing.

## 3. Advanced Lipid Technologies® (ALT®)

The optimal composition of lipophilic substances and surfactants is crucial to enhance solubility, increase lipid absorption, reduce food effect, and achieve consistent bioavailability [1, 2]. For the past 10 years, Micelle BioPharma, Inc. (former Sancilio & Company, Inc. or SCI) has been working on the development and refinement of a proprietary lipid delivery platform that forms a stable, uniform dispersion under a range of standard conditions that are commonly seen during dosing of an oral drug product. This lipid delivery system, known as Advanced Lipid Technologies® or ALT®, promotes lipid digestion and absorption even in the absence of bile salts [3–5].

Micelle BioPharma has used ALT® to improve the bioavailability of various lipophilic compounds such as fatty acids and hormones. This paper will focus on the use of ALT® to improve DHA and EPA bioavailability, two omega-3 fatty acids with a significant impact on several human disorders, as a model for this delivery platform. These formulations contain highly purified DHA and EPA ethyl esters as the API in a surfactant system composed of the nonionic surfactants, polysorbate 80 (polyoxyethylene (20) sorbitan monooleate), and the nonionic, triblock copolymer surfactant, poloxamer 237 (polyoxyethylene-polyoxypropylene block copolymer). The specific formulations that have been developed are described in detail below.

(i)* SC401.* This formulation uses Omega-3-Acid Ethyl Esters, USP as the API, which meets the quality attributes of the USP monograph 6. The omega-3 oil contains between 430 and 495 mg/g of eicosapentaenoic acid ethyl ester (EPA-EE) and between 347 and 403 mg/g of docosahexaenoic acid ethyl ester (DHA EE) and no less than 90% w/w of total omega-3 fatty acid ethyl esters. This formulation, in soft gelatin capsules, is currently under development.

(ii)* SC411/SC403.* This formulation consists of highly purified, DHA EE as the API (≥93%). This formulation may contain small amounts of EPA-EE and the other omega-3-acid ethyl esters, i.e., eicosatetraenoic acid, docosapentaenoic acid, stearidonic acid, alpha-linolenic acid, and heneicosapentaenoic acid, normally found in fish oil. The soft gelatin capsule dosage form of this API has been named SC411, and the liquid formulation has been named SC403. The different dosage forms (SC411 or SC403) of the same formulation were designed based on the different applications of the drug as explained in the subsequent sections of this paper.

**Figure 1:**
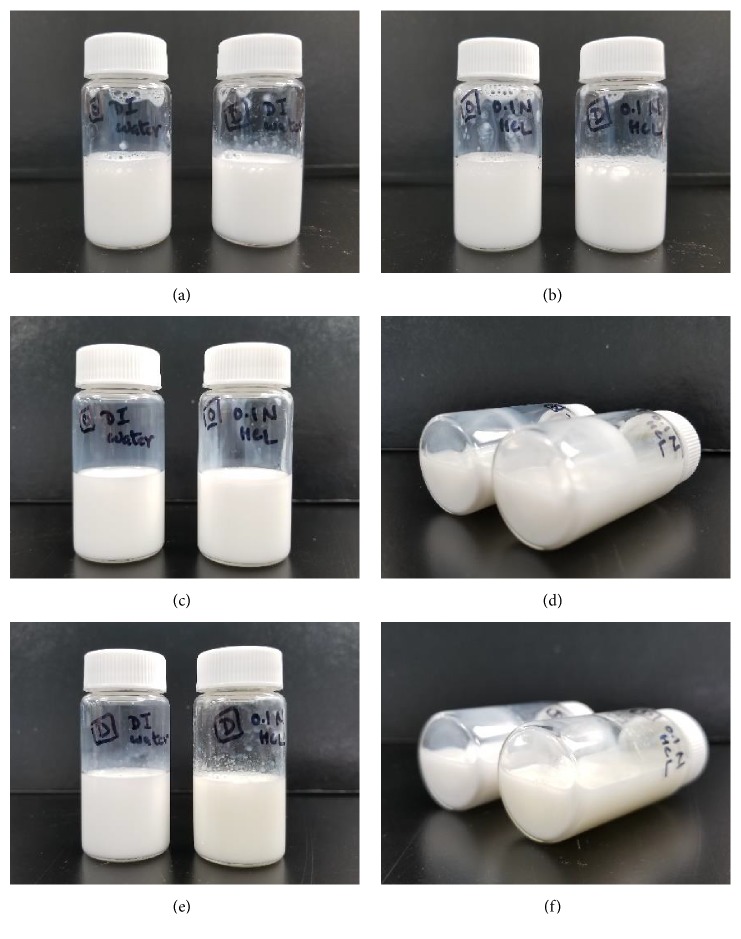
*Physical Stability of formulations of SC401 and SC411/SC403 dispersed in purified water and 0.1 N HCl*. (a) SC401 (left) and SC411/SC403 (right) formulations forming micelles in a 1:20 dilution in purified water at time 0, (b) SC401 (left) and SC411/SC403 (right) formulations forming micelles in a 1:20 dilution in 0.1 N HCl at time 0, (c) SC401 formulation forming micelles in a 1:20 dilution in purified water (left) and 0.1 N HCl (right) 7 days after dilution, (d) SC401 formulation forming micelles in a 1:20 dilution in purified water (left) and 0.1 N HCl (right) 7 days after dilution in a horizontal position, showing no precipitates of the two-phase formation, (e) SC411/SC403 formulation forming micelles in a 1:20 dilution in purified water (left) and 0.1 N HCl (right) 7 days after dilution, and (f) SC411/SC403 formulation forming micelles in a 1:20 dilution in purified water (left) and 0.1 N HCl (right) 7 days after dilution in a horizontal position, showing no precipitates of the two-phase formation.

**Figure 2:**
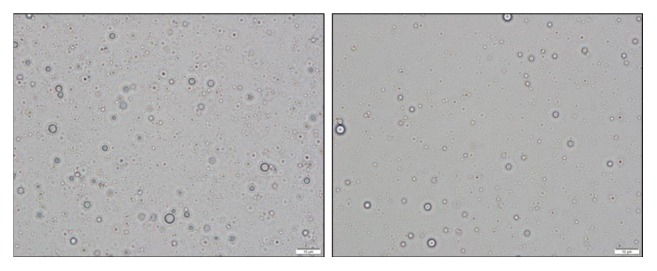
Micrographs illustrating dispersions of SC401 (left) and SC411 (right) at a 1:20 dilution in purified water. Taken with a BX43 Olympus Light Microscope using cellSens software for image capture and particle size measurement (1000X magnification).

For the two formulations detailed above, the ratio of omega-3 oil, polysorbate 80, and poloxamer 237 was optimized by evaluating the ability to spontaneously form uniform and stable micelles in both purified water and 0.1 N HCl, pH 1.2. Consistent with other self-dispersing systems 7-16, this formulation spontaneously forms a micellar emulsion with little added energy when added to an aqueous medium and stays stable for long periods of time (Figure 1). Micrographs of the dispersed micelles for each formulation are shown in Figure 2. Laser diffraction particle size analysis of the micelles formed from SC401 and SC411/SC403 are summarized in Table 1. For these studies, Single Particle Optical Sensing (SPOS) was used to characterize the globule size distribution from approximately 0.5 micron to 500 microns. The micelles that are formed have a mean of approximately 1-2 micrometers for both formulations. Polysorbate 80 is the primary emulsifier in this system and presumably makes up the bulk of the surface of the micelle as it is present in excess to poloxamer 237. It is postulated that poloxamer 237 strengthens and stabilizes the micelle structure by insertion into the oil-water interface that is formed by polysorbate 80. This mechanism has been observed and reported with other poloxamers in lipid monolayer systems 17-21.

## 4. Effect of ALT® on Fat Absorption: Clinical and Preclinical Studies

The ALT®-containing formulations described in this paper spontaneously form a stable micellar emulsion even in the absence of bile salts. This mechanism greatly increases the surface area of the omega-3 oil, presenting the oil as the equivalent to finely emulsified micelles. This allows for quick digestion of the omega-3 oils by lipases and for achieving consistent omega-3 absorption and BA, independent of it being dosed with a fatty meal [36].

The ability of ALT® to improve omega-3 fatty acid absorption makes these formulations potentially beneficial for their clinical application. During preclinical studies, both SC401 and SC403/SC411 formulations were evaluated for oral toxicity in rats. Based on 28-day and 90-day juvenile rat toxicity studies that were conducted with these formulations, the no observed adverse effect level (NOAEL) is 2000 mg DHA EE/kg/day for oral administration. They have a low potential for general toxicity, genotoxicity, and developmental toxicity and therefore can be expected to have an overall benign safety profile.

In the following section, we describe some of Micelle BioPharma's sponsored clinical development programs:

### 4.1. SC401 for the Treatment of Hypertriglyceridemia

**Figure 3:**
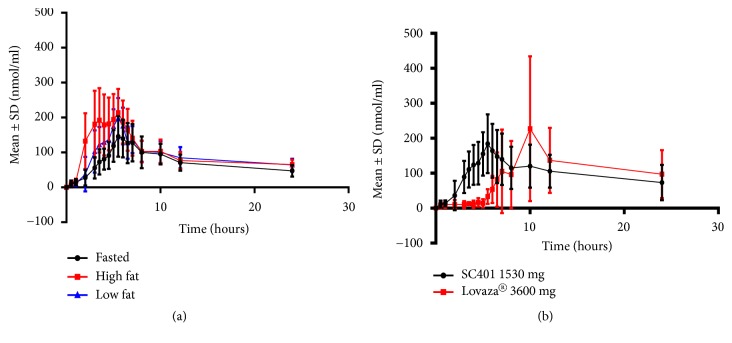
*Advanced Lipid Technologies® (ALT®) significantly improves omega-3 fatty acid absorption*. (a) Phase I, Single-Dose, Open-Label, Randomized, Three-Way Crossover Study of Food Effect on the Pharmacokinetics of Omega-3-Acid Ethyl Esters in Healthy Subjects was performed to quantify the BA of EPA + DHA after treatment with 1530 mg EPA EE+ DHA EE in fasted and high or low fat feeding conditions. Mean ± standard error of the total EPA + DHA concentration-time profiles after a single oral dose of SC401 (1530 mg of DHA EE + EPA EE with ALT®) in fasted, low fat or high fat diet conditions. (b) Phase I, Single-Dose, Open-Label, Randomized, Two-Way Crossover Study, the Relative Bioavailability of 1530 mg of EPA EE + DHA EE with ALT® vs. 3600 mg of EPA EE + DHA EE, was compared under low fat fed conditions. Mean ± standard deviation of total lipid EPA + DHA concentration-time profiles after a single oral dose of SC401 (1530 mg of DHA-EE + EPA EE) or Lovaza® (3600 mg of DHA EE + EPA EE) in low-fat feeding conditions (N = 23). DHA = docosahexaenoic acid; EPA = eicosapentaenoic acid.

As explained before, SC401 represents a new formulation of EPA EE and DHA EE using ALT® for the purpose of reducing the food effect and improving EPA EE and DHA EE bioavailability for the treatment of hypertriglyceridemia. Most patients prescribed omega-3 oils suffer from elevated triglycerides and high fat meals should be avoided. On the other hand, the currently marketed omega-3 EE drug products containing ethyl esters have to be dosed with a fatty meal to minimize the negative food effect. When these drug products are dosed in a fasting condition, relatively little of the drug gets absorbed [10]. The SC401 formulation has proven to be efficient in increasing the BA and significantly improving absorption of EPA EE and DHA EE even in low-fat or fasting conditions. A Phase I, Single-Dose, Open-Label, Randomized, Three-Way Crossover Study of Food Effect on the Pharmacokinetics of Omega-3-Acid Ethyl Esters in Healthy Subjects was performed to quantify the BA of EPA + DHA after treatment with 1530 mg EPA EE + DHA EE in fasted and high- or low-fat feeding conditions. This study showed a high BA for DHA + EPA, even in fasting conditions (Figure 3(a)) [36]. In another Phase I, Single-Dose, Open-Label, Randomized, Two-Way Crossover Study, the relative bioavailability of two SC401 capsules (1530 mg of EPA EE + DHA EE with ALT®) was compared with four Lovaza® capsules (3600 mg of EPA EE + DHA EE) under low fat feeding conditions (Figure 3(b)) [37]. Two capsules of SC401 produced a baseline adjusted Cmax of approximately 203 nmol/mL and an area under the curve (AUC0–last) of approximately 2323 h nmol/mL for total EPA + DHA, which were relatively biocomparable with those achieved by four capsules of Lovaza®, with a baseline-adjusted Cmax of approximately 252 nmol/mL and an AUC0–last of approximately 2363 h nmol/mL. In addition, SC401 showed a significantly lower Tmax compared with Lovaza®. These results indicate that the SC401 formulation produced similar omega-3 fatty acid levels in the blood with less than half the dosage of Lovaza, and it also increased the speed of omega-3 fatty acid entry into the circulation. This study also showed a lower intersubject variability for SC401 versus Lovaza® for both Cmax and AUC0-last, as reflected in the % mean geometric coefficient of variance[37], suggesting that ALT® induces a more reproducible, consistent and predictable BA that is less dependent on external factors like the amount of fat in food.

**Figure 4:**
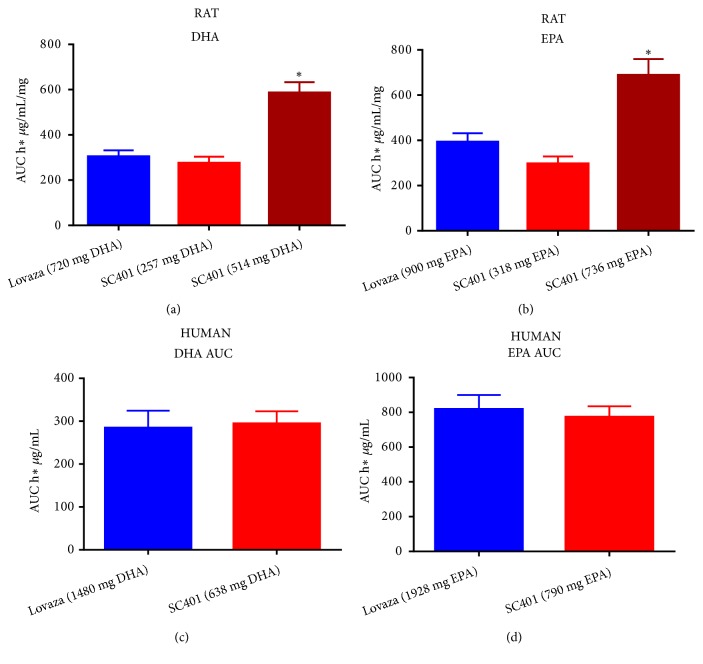
*SC401 significantly increases DHA and EPA bioavailability in both rats (a, b) and humans (c, d) compared with Lovaza®*. (a) Comparison of the area under the curve (AUC) of DHA at time 0 after treating adult rats with 2000 mg/kg of Lovaza® (720 mg DHA EE, blue), 1000 mg/kg of SC401 (257 mg DHA EE, red), or 2000 mg/kg of SC401 (514 mg DHA EE, dark red). (b) Comparison of the area under the curve (AUC) of EPA at time 0 after treating adult rats with 2000 mg/kg of Lovaza® (900 mg EPA EE, blue), 1000 mg/kg of SC401 (318 mg EPA EE, red), or 2000 mg/kg of SC401 (736 mg EPA EE, dark red). (c) Comparison of the AUC of DHA at time 0 after treating with Lovaza® (1480 mg of DHA EE, blue) or SC401 (638 mg DHA EE, red), in healthy humans. (d) Comparison of the AUC of EPA at time 0 after treating with Lovaza® (1928 mg EPA EE, blue) or SC401 (790 mg EPA EE, red), in healthy humans. *∗* p<0.001 vs Lovaza® and higher dose of SC401.

Our studies, performed in both rats and humans, show how the SC401 formulation greatly improves EPA-EE and DHA EE BA and mitigates the food effect associated with their absorption (Figure 4) [36, 37]. This formulation should allow for a reduction of the required dose of omega-3 fatty acids, while providing reproducible results, with less variability and eliminating the need to dose in conjunction with high fat meals.

### 4.2. SC403 for the Treatment of Short Bowel Syndrome

SC403 is an oral liquid formulation of DHA-EE using ALT® that was designed to increase the absorption of DHA in infants and children with Short Bowel Syndrome (SBS).

SBS is a condition that occurs when a large segment of bowel is completely dysfunctional or has been surgically removed. Diarrhea is the main symptom of short bowel syndrome leading to dehydration, malnutrition, and weight loss. The disorder usually does not develop unless more than two thirds of the small intestine is missing or dysfunctional. In that case, specific nutrients are not properly absorbed into the body (malabsorption) with fats and fat-soluble nutrients being especially poorly absorbed. This makes the patients highly dependent on parenteral nutrition (PN), with its associated risk of liver damage [38]. SBS is particularly severe in newborn children, because of their increased need of nutrients for their fast relative growth and development. Particularly, the deficit of omega-3 fatty acids, mostly DHA, in perinatal brain development has been associated with neurological and psychological disorders [39]. Omega-3 fatty acids have been demonstrated to improve the outcome of patients with damaged liver, particularly nonalcoholic fatty liver disease and parenteral-induced liver injury, which make them a potential therapeutic agent for patients with SBS [40–44].

By virtue of its formulation and mechanism of action, it is hypothesized that SC403 increases fatty acid absorption in patients with SBS, ameliorating the malabsorption in these patients and providing the benefits previously explained of omega-3 fatty acids. The use of ALT® facilitates micelle formation in the gut and significantly increases DHA bioavailability as previously described.

**Figure 5:**
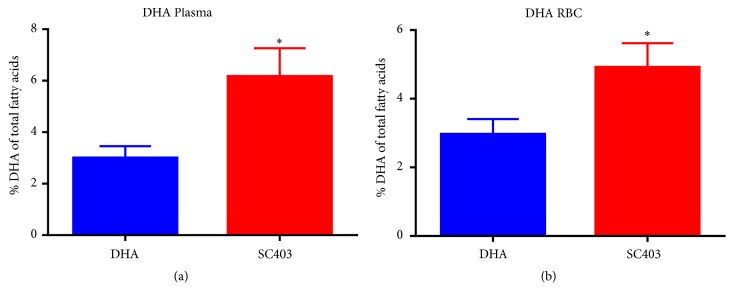
*SC403 significantly increases DHA levels in both plasma and red blood cells (RBC) membrane in a piglet model of Short Bowel Syndrome (SBS)*. After a 70% mid-jejune-ileal resection, neonatal piglets were fed with 1 g/kg/day of DHA EE or 1 g/kg/day of DHA EE + ALT® (SC403) and the percentage of DHA was measured in blood. The comparison of DHA plasma levels in DHA (blue) vs. SC403 (red) treated SBS piglets reveals a statistically significant increase in DHA in SC403-treated piglets, both in plasma (a) and red blood cells membranes (b) 8 days after intervention (SEM ± SE) as compared with the piglets treated with DHA alone. *∗*: p<0.005.

The study results shown in Figure 5, using a porcine animal model for SBS, demonstrate the efficacy of SC403 in increasing DHA absorption and its incorporation into red blood cell (RBC) membranes as compared with a DHA control formulation without ALT® [45]. It is plausible to conclude that the use of SC403 will increase enteral essential fatty acid absorption that is critical for growth and development in pediatric short bowel patients. It has been postulated that treatment with SC403 will eventually lead to a decrease in PN requirements for SBS patients, thus reducing the risk of associated liver injury (cholestasis).

### 4.3. SC411 for the Treatment of Sickle Cell Disease

As explained previously, SC411 is an oral formulation of DHA-EE using ALT® presented in soft gelatin capsules that was designed to increase the absorption of DHA and other essential fatty acids in Sickle Cell Disease (SCD) patients.

SCD is a hereditary blood disorder, due to an abnormality in the oxygen-carrying hemoglobin molecule in RBCs. SCD is characterized by a change in the shape of erythrocytes (red blood cells) from their normal “doughnut like” form to a “sickle like” (crescent) form. Sickle cells are stiff and sticky and tend to block blood flow in the blood vessels of the limbs and organs, causing what is known as a sickle cell crisis: obstructed capillaries (causing ischemia, pain, necrosis, and organ damage), splenic sequestration (causing pain and increased risk of infection), acute chest syndrome (causing chest pain, fever, and pulmonary symptoms), aplastic crisis (causing baseline anemia and pallor, tachycardia, and fatigue), and hemolytic crisis (causing a decrease of erythrocytes). SCD has no widely available cure. The available treatments are directed to relieve pain, prevent infections, organ damage, and strokes, and control complications. Recent studies have shown that SCD patients have abnormally low levels of DHA in their RBC membranes [46], and the treatment with DHA increases red blood cell membrane flexibility and fluidity [47] and reduces the number of crises in SCD patients [48]. For these reasons, the SC411 formulation may correct the DHA levels in erythrocytes and other blood cells and potentially ameliorate SCD-related acute and chronic complications.

**Figure 6:**
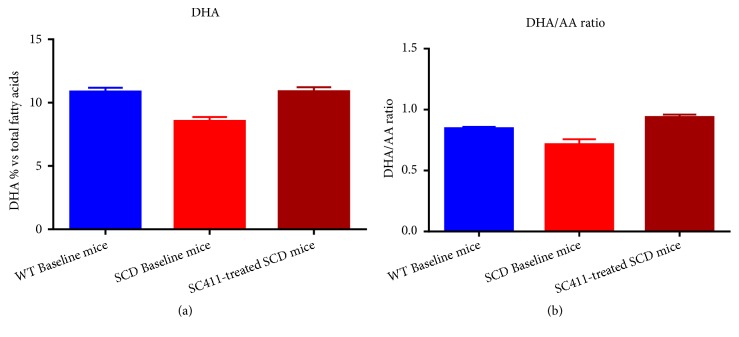
*SC411 significantly increases DHA levels in red blood cell (RBC) membranes in a model of Sickle Cell Disease (SCD)*. Wild type (WT) and Townes, a recognized animal model of SCD, mice with a deficiency in DHA were treated with SC411 (50 mg DHA/Kg/day + ALT®) for 42 days before measuring the RBC membrane levels of DHA. (a) The SCD mice (red) have significantly lower levels of DHA as compared with WT (blue) at baseline. SC411-treated SCD mice (dark red) restore the RBC membrane DHA levels to WT levels. (b) Similarly, the DHA/AA ratio in RBC membranes is also low in SCD mice (red) as compared with WT (blue) at baseline and SC411-treated SCD mice (dark red) significantly increase DHA/AA ratio in RBC membranes.

Consistent with what has been previously described in humans [49], the Townes mouse model of human SCD showed significantly lower baseline DHA levels and DHA/arachidonic acid (AA) ratio as compared to wild type (WT). After three weeks of treatment with SC411, the SCD mice displayed increased DHA to a level comparable to that observed in WT mice (Figure 6).

**Figure 7:**
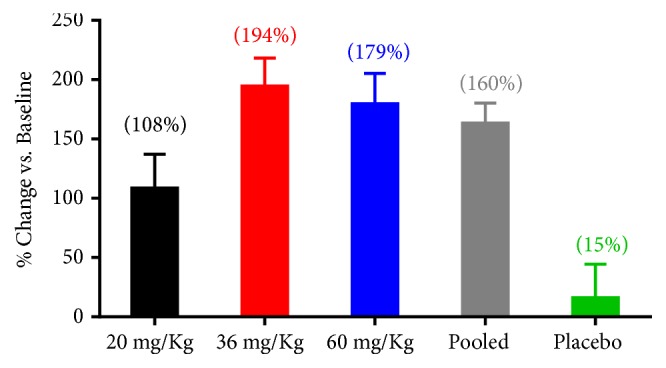
*Percent change of DHA + EPA blood cell membrane levels from baseline at week 4*: after four weeks of treatment (A), blood cell membrane DHA and EPA levels were significantly increased in all SC411 doses (p<0.001) vs. baseline. 36 mg/Kg, 60 mg/Kg, and pooled treatments were also significantly increased vs. placebo (p<0.01).

The ability of SC411 to significantly increase DHA levels in RBC was also proven in the Sickle Cell Omega-3 Treatment Trial (SCOT Trial). In this phase 2, dose finding study, the treatment of sickle cell patients for 30 days with three different doses of SC411 induced a significant increase in DHA incorporation in blood cells (p<0.001, Figure 7) and significantly positive feedback in specific biomarkers related with SCD [50].

## 5. Conclusions

Lipophilic compounds are hydrophobic substances that are highly insoluble in aqueous media and are therefore poorly absorbed into the body. Particularly, omega-3 fatty acids play a key role in mammalian metabolism. The absorption of omega-3 fatty acids in the intestine can be compromised by several factors, including food effect or malabsorption associated with different medical conditions and disease states. In addition, there are several conditions in which there is a need for replenishing an omega-3 fatty acid deficiency, like the case of sickle cell disease, so there is a need for new technologies that could improve lipid absorption and BA. The use of ALT® in different formulations and dosage forms developed by Micelle BioPharma has been demonstrated to greatly improve omega-3 fatty acid BA. The ability of ALT® to increase the BA of lipophilic substances may help reduce the required oral dose, minimize inter- and intraindividual BA variability, and optimize the potential therapeutic effects of a potentially wide range of lipophilic drugs.

## Figures and Tables

**Table 1:** *Globule size distribution of SC401 and SC411/SC403 in purified water*. Samples were prepared by dispersing formulations in purified water. Results displayed above for D10, D50 and D90 are percentage of particles less than the indicated size on number basis.

Formulation	Technique	Cumulative Number Distribution			
		Mean	D10	D50	D90
SC401	Single Particle Optical Sensing (SOPS)	2.08 *μ*m	0.58 *μ*m	0.84 *μ*m	4.13 *μ*m
SC411/SC403		0.71 *μ*m	0.53 *μ*m	0.63 *μ*m	0.95 *μ*m
